# Study on the synthesis mechanism of sodalite, gismondine, and zeolite-P1 zeolite materials from ladle furnace slag and fly ash

**DOI:** 10.1038/s41598-023-30282-y

**Published:** 2023-02-24

**Authors:** Wenqing Ma, Yuanrong Yi, Minghang Fang, Chunhui Li, Jie Li, Wei Liu

**Affiliations:** 1 College of Ecology and Environment, Xinjiang University, Urumqi, 830046 People’s Republic of China; 2 Key Laboratory of Oasis Ministry of Education, Xinjiang University, Urumqi, 830046 People’s Republic of China; 3 Key Laboratory of Smart City and Environmental Modeling Autonomous Region, Urumqi, 830046 People’s Republic of China

**Keywords:** Environmental sciences, Metal-organic frameworks

## Abstract

In this study, geopolymers were prepared using ladle furnace slag (LFS) and fly ash (FA), and hydrothermal treatment was then used to synthesize bulk zeolite molecular sieves with gismondine, zeolite-P1, and sodalite phases. The effect of the synthesis conditions on the crystalline phases of the zeolite molecular sieves was investigated by XRD. The results showed that the best zeolite molecular sieves were prepared with an LFS: FA ratio of 4: 6, a curing temperature of 40 °C, a curing time of 12 h, a sodium silicate modulus (Ms) of 1.4, a NaOH concentration of 4 mol/L, a hydrothermal temperature of 120 °C, and a hydrothermal time of 12 h. On this basis, the products were analyzed by SEM, N_2_ adsorption, and FT-IR. The results showed that the synthesized zeolite molecular sieves had mesoporous properties, and the degree of polymerization and cross-linking of the silica-aluminate gel were enhanced after hydrothermal treatment. In addition, the formation mechanism of the zeolite molecular sieves was explored through the changes of the silica-alumina during zeolite formation. This paper is the first to use the hydrothermal conversion of zeolite molecular sieves from LFS-FA based polymers to provide some guidance for the resource utilization of LFS and FA.

## Introduction

Ladle furnace slag (LFS) and fly ash (FA) are two of the main solid waste products emitted by the steel industry and the coal-fired power generation industry^1–3^. About 20–25 billion tons of solid waste (such as waste rock, sludge, and slag) and about 5–7 billion tons of tailings are produced worldwide every year^1,4^. Meanwhile, the largest manufacturers in China, India, and the United States produce at least 800 million tons of FA every year, but only 20% of this FA is used as additives for cement and concrete-related applications^5^. If LFS and FA are not properly dealt with, waste will accumulate, leading to potentially severe impacts and immeasurable harm to the environment^6^. Therefore, an effective method for utilizing bulk LFS is urgently needed. The preparation of geopolymers from solid wastes is an economical, safe, and environmentally friendly strategy that might be useful for this purpose. Moreover, reusing and adding value to solid waste are also in line with the Sustainable Development Goals (SDGs) of the United Nations and the implementation of the Paris Agreement. Because zeolites are widely used in adsorption, ion exchange, catalysis, molecular sieve, and other applications^7–10^, it is estimated that the global market for synthetic zeolites in 2023 will reach about 5.9 billion dollars^11^. Geopolymers composed of [SiO_4_]^4−^ and [AlO_4_]^5−^ tetrahedra are generally regarded to be the amorphous prepolymers of crystalline zeolites^12,13^. Zeolites can be prepared by hydrothermal methods, and these hydrothermally prepared zeolites have exhibited better crystal structures and better performance for fixing heavy metal ions. At the same time, zeolites with specific macroscopic structures also have broad application prospects in solid waste recycling. Cheng et al.^14^ extracted SiO_2_ from nickel–iron slag and prepared 4A zeolite by hydrothermal method, reporting that the optimal hydrothermal conditions were a hydrothermal temperature of 100 °C and a hydrothermal time of 8 h. Liu et al.^15^ used ultrafine circulating fluidized bed FA as a raw material to prepare A-type zeolite by alkali-activated hydrothermal synthesis method, and their optimal synthesis conditions were an alkali concentration of 2.6 mol/L, a hydrothermal temperature of 90 °C, and a hydrothermal time of 6 h.

However, due to the high calcium content of LFS, there are few reports on the synthesis of zeolites using LFS as a silicon and aluminum source. It is generally believed that when materials with high calcium content are converted to zeolites, calcium silicate species or hydroxysodalite with low porosity and low cation exchange capacity are formed, which inhibits the formation of the zeolite^12,16^. To convert high calcium-based raw materials into zeolites, specific pretreatment or synthesis methods are usually required. For example, Murakami et al.^17^ used citric acid and formic acid solutions to selectively elute calcium from blast furnace slag three times in a ball-milling reactor and then synthesized zeolite A from the residue. Park et al.^18^ prepared zeolite materials containing sodalite by treating FA, NaOH, and NaNO_3_ at 350 °C for 24 h, washing the obtained solids with excessive deionized water at least seven times, and drying the product overnight at 105 °C. Lei et al.^19^ used dispersed-suspension-curing technology to fabricated metakaolin/slag-based zeolite microspheres. These spheres were then cured in an oven at 85 °C for 24 h, filtered, cleaned, dried at 120 °C for 8 h, and finally calcined at 500 °C for 3 h to obtain zeolite microspheres.

These previous studies have reported the successful synthesis of zeolites, but the reported methods have the disadvantages of long process times, complex operation, low solid waste utilization rates, high economic cost, and the production of acid solution by acid leaching will cause a burden on the environment. Therefore, a simple and environmentally friendly method is needed to synthesize zeolites from solid waste. In this study, a geopolymer precursor was synthesized from high calcium-based LFS and FA for the first time, and the effect of different synthesis conditions on the prepared zeolite molecular sieves was studied by the in situ hydrothermal conversion of the geopolymer into massive zeolite molecular sieves to obtain the best preparation conditions. Based on the optimal conditions, the microstructure and morphology of the products were studied, and the formation mechanism of the zeolite was described. This process is characterized by a short processing time, low energy consumption, simple operation, environmental friendliness, and easy recycling due to the macro morphology of the zeolite.

## Materials and methods

### Raw materials

**Figure 1:**
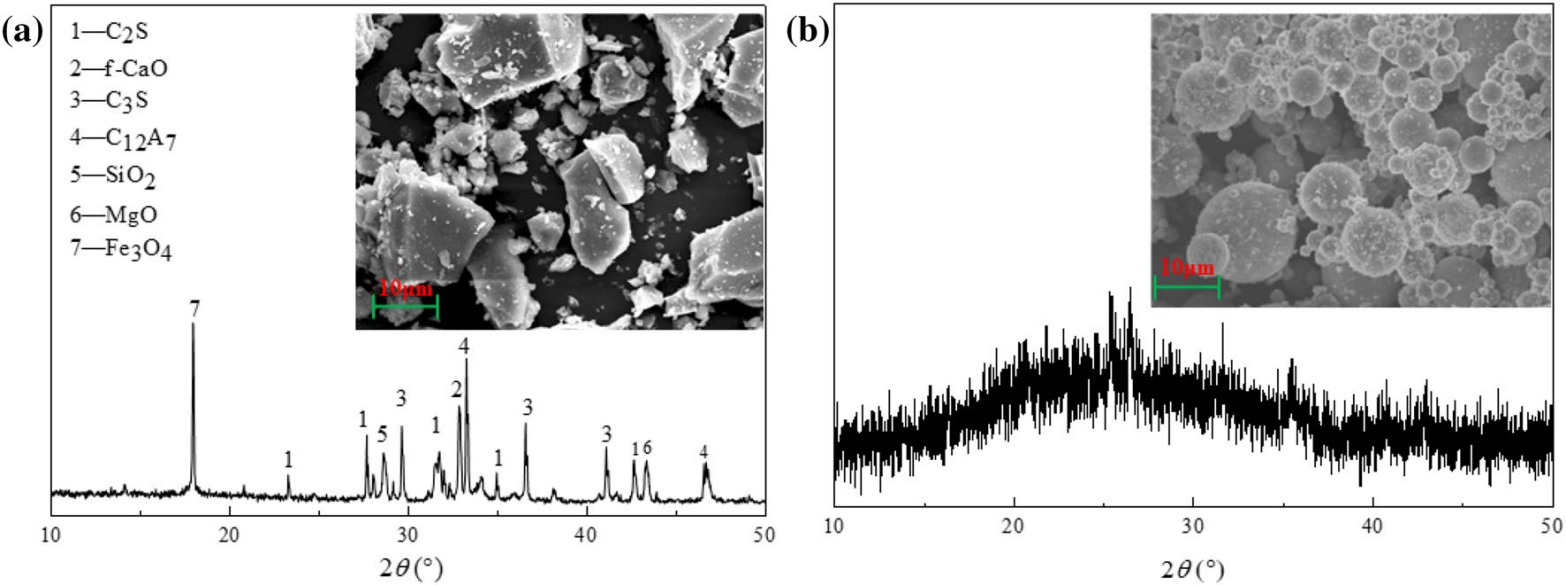
XRD and SEM imagines of the raw LFS (**a**) and raw FA (**b**); C_3_S: Ca_3_SiO_5_, C_12_A_7_: 12CaO ∙ 7Al_2_O_3_.

The LFS in this experiment was obtained from a steel plant in Xinjiang, China, and the FA was obtained from a power plant in Xinjiang, China. The chemical composition of these materials is shown in Table 1. The main components of LFS were CaO, SiO_2_, and Al_2_O_3_, while the main components of FA were SiO_2_, Al_2_O_3_, and CaO. XRD and SEM patterns are shown in Fig. 1. The main mineral phase composition of the LFS is f-CaO, C_2_S etc., with irregular shape and smooth surface. The mineral phase composition of FA is mainly non-crystalline with a spherical morphology. Alkali activator: An appropriate amount of granular sodium hydroxide and deionized water were added to industrial grade sodium silicate with a modulus (modulus: Ms = nSiO_2_: Na_2_O) of 3.26, and a modified sodium silicate solution was obtained after mixing. The sodium hydroxide was analytically pure.

**Table 1:** Chemical composition of LFS and FA(wt.%).

Specimen	CaO	SiO_2_	Al_2_O_3_	Fe_2_O_3_	MgO	SO_3_	Na_2_O	TiO_2_	MnO	LOI
LFS	59.39	16.89	10.12	4.62	2.91	1.77	–	1.18	0.85	2.27
FA	10.36	48.09	18.48	8.40	3.96	2.20	5.15	0.94	0.14	2.28

### Synthesis of zeolite molecular sieve blocks

After evenly stirring the LFS and FA, the modified sodium silicate solution was added. This mixture was poured into a mold, shaken until the surface was free of bubbles, and placed into an oven for several hours. After demolding, the block was loaded into a 100 mL polytetrafluoroethylene thermal reactor, and the sodium hydroxide solution was added to fill 2/3 of the container. After hydrothermal treatment, the product was cooled at room temperature and washed to neutral with deionized water, then dried to obtain a zeolite molecular sieve block. The experimental conditions are summarized in Table 2.

**Table 2:** Synthesis conditions for LFS and FA.

Sample	Ratio of LFS to FA	NaOH concentration (mol/L)	Ms	Curing treatment		Hydrothermal treatment	
				t/h	T/°C	t/h	T/°C
LF 1-4	7:3	4	1.4	12	40	12	120
	6:4						
	5:5						
	4:6						
NC 1-5	4:6	1	1.4	12	40	12	120
		2					
		3					
		4					
		5					
Ms 1-4	4:6	4	1.0	12	40	12	120
			1.2				
			1.4				
			1.6				
Ct 1-4	4:6	4	1.4	6	40	12	120
				12			
				18			
				24			
CT 1-4	4:6	4	1.4	12	40	12	120
					60		
					80		
					100		
Ht 1-4	4:6	4	1.4	12	40	6	120
						12	
						18	
						24	
HT 1-4	4:6	4	1.4	12	40	12	60
							90
							120
							150

### Sample characterization

The mineralogical compositions of the specimens were determined using X-ray diffractometry (XRD, BrukerAXS D8, Germany), and the XRD spectra were recorded using Cu K radiation and a 2θ range of 10 to 80°. The chemical composition of the samples was determined using X-ray fluorescence (XRF, Thermo Scientific ARL Perform'X, America). The morphology and microstructure of the prepared specimens were examined by scanning electron microscopy (SEM, ZEISS Sigma 300, America) at an accelerating voltage of 15 kV. N_2_ adsorption–desorption experiments were performed at 77 K using an automatic ASAP 2460 (Micromeritics ASAP 2460, America). The pore volumes and pore size distributions were calculated by the Barrett-Joyner-Halenda (BJH) method, BJH pore size distribution is divided into an adsorption pore size distribution and a desorption pore size distribution. Fourier transform infrared (FT-IR) spectra of the specimens were obtained by an FT-IR spectrometer (Thermo Scientific Nicolet iS20, America) using the KBr pellet dics method. Each sample was tested at a resolution of 2 cm^−1^ with 32 scans. The blank KBr pellet was tested at the sametime as reference.

## Results and discussion

### Influence of synthesis conditions on zeolite

#### Ratio of LFS to FA

**Figure 2:**
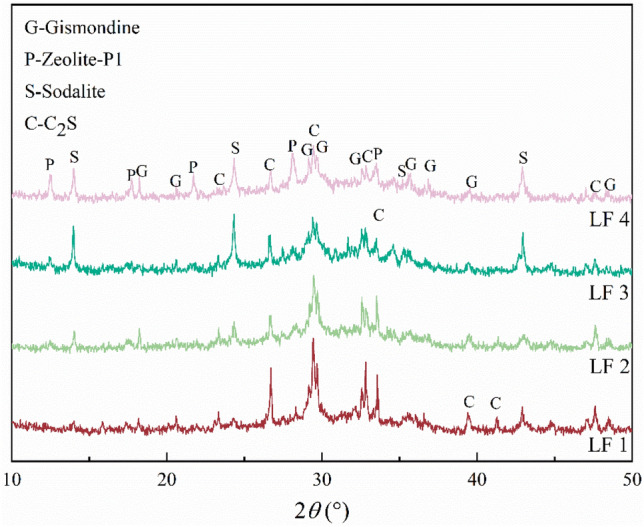
XRD patterns of hydrothermal products with different mass ratio of LFS to FA; LF 1:the mass ratio of LFS to FA is 4:6, LF 2: the mass ratio of LFS to FA is 5:5, LF 3: the mass ratio of LFS to FA is 6:4, LF 4: the mass ratio of LFS to FA is 7:3.

The XRD patterns of zeolites prepared using different mass ratio of LFS to FA are shown in Fig. 2. Diffraction peaks attributed to four different phases were observed after hydrothermal treatment. These phases were gismondine (Ca_2_Al_4_Si_4_O_16_·9H_2_O), zeolite-P1 (Na_6_Al_6_Si_10_O_32_·12H_2_O), sodalite (Na_8_Al_6_Si_6_O_24_), and C_2_S (Ca_2_SiO_4_). The gismondine peak intensity increased with increasing FA content, the diffraction peak of zeolite-P1 appeared when LFS:FA was 4:6 (LF 4), and the peak intensity of sodalite increased with increasing FA content. The C_2_S peak increased in intensity when LFS:FA was 7:3(LF 1) but decreased in intensity with increasing FA content. The formation of gismondine was potentially due to the high CaO content (59%) in the LFS. In the geopolymer preparation process, CaO was transformed into a calcium-rich silicon phase. The subsequent hydrothermal treatment caused the calcium-rich silicon phase in the geopolymer to dissolve into the liquid phase under the action of NaOH. Thus, the Ca^2+^ in the liquid phase was integrated into the lattice of some of the zeolite, leading to the formation of gismondine. When the content of LFS was too high, the active Si–Al in the system was insufficient to form the zeolite-P1 phase; meanwhile, the LFS with high CaO content preferentially formed gismondine and calcium silicate to inhibit the formation of zeolite-P1^12,16,19^. Finally, with increasing FA addition, the active Si–Al content in the system increased. This also encouraged the formation of more silica-aluminate gels, which were subsequently converted to zeolite phases. The decline in the C_2_S peak intensity with increasing FA content may be because the FA was rich in Si and Al. With the incorporation of FA, the Si and Al content in the system increased. Some Si and Al species released by FA participated in the formation of various zeolites, and because the C_2_S was unstable, it was easily occupied by Si. This resulted in the formation of more stable zeolite-P1, gismondine, and sodalite.

#### NaOH concentration

**Figure 3:**
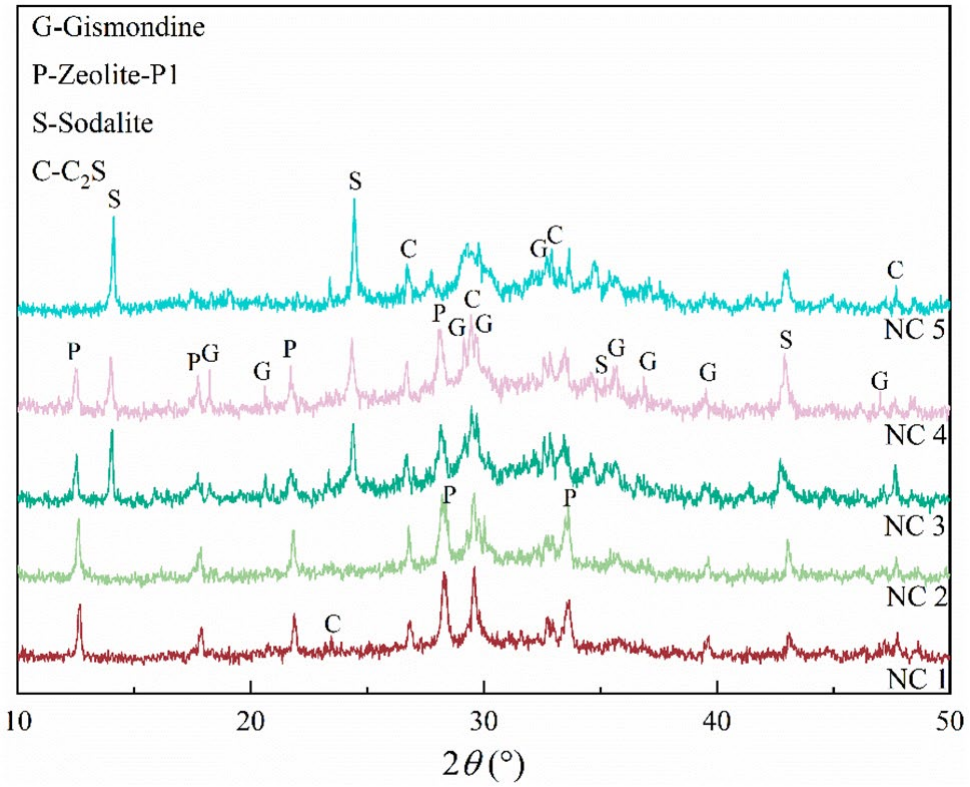
XRD patterns of hydrothermal products with different NaOH concentration; NC 1: the NaOH concentration is 1 mol/L, NC 2: the NaOH concentration is 2 mol/L, NC 3: the NaOH concentration is 3 mol/L, NC 4: the NaOH concentration is 4 mol/L, NC 5: the NaOH concentration is 5 mol/L.

As the main driving force for depolymerizing geopolymers and rearranging silica-aluminate ions and zeolites, NaOH severely affects the structure and morphology of zeolites^20^. An excessively low NaOH concentration cannot depolymerize geopolymers, and metal cations therefore cannot enter the lattice^21^. In contrast, an excessively high NaOH concentration leads to the dissolution and recrystallization of zeolite particles. Figure 3 shows the XRD patterns of zeolites prepared with different NaOH concentration. With increasing NaOH concentration, the diffraction peak of zeolite-P1 exhibited a 0.1° shift to the left when the NaOH concentration was 3 or 4 mol/L (NC 3 or NC 4). This peak disappeared and sodalite was observed when the NaOH concentration was 5 mol/L (NC 5), and the diffraction peak of gismondine also disappeared. Compared with zeolite-P1, sodalite contains more Na^+^ and Al^3+^ ions. In this synthesis, more Na^+^ was provided with increasing NaOH concentration. The presence of Na^+^ can balance the negative charges in the system and provide a structural guidance function^22^. Therefore, the transformation of zeolite-P1 to sodalite was observed at a NaOH concentration of 5 mol/L . This result is similar to those of other researchers^23^, who have reported that sodalite can be obtained at high alkali concentrations and zeolite-P1 can be obtained at low alkali concentrations. In addition, a high alkali concentration can also improve the leaching efficiency of Al_2_O_3_, accelerate crystal growth, and shorten the formation time of crystal nuclei^23^. The diffraction peaks of gismondine were similar to those of zeolite-P1. This was potentially because gismondine is composed of four-membered and eight-membered rings, but the number of aluminum-oxygen tetrahedra in these rings is low, so gismondine is only stable in low concentration media^24^.

#### Sodium silicate modulus (Ms)

**Figure 4:**
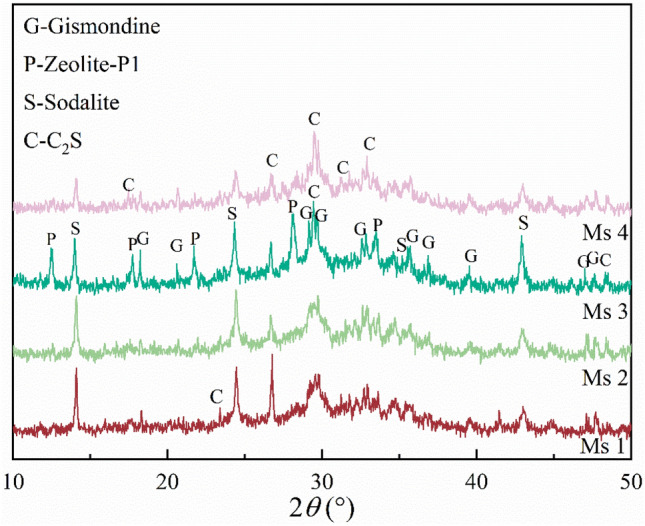
XRD patterns of hydrothermal products with different sodium silicate modulus (Ms) value; Ms 1: sodium silicate modulus is 1.0, Ms 2: sodium silicate modulus is 2.0, Ms 3: sodium silicate modulus is 3.0, Ms 4: sodium silicate modulus is 4.0.

Figure 4 shows the XRD patterns of zeolites prepared with different sodium silicate modulus (Ms) values. The modulus of sodium silicate, largely determines the structure of the polymer in the polymerization state of silica-aluminate ions, which affects the structure of the resulting zeolite^20,25,26^. When Ms was 1.0 or 1.2 (Ms 1 or Ms 2), only a small amount of sodalite was generated but when Ms was 1.4 (Ms 3), the diffraction peak of zeolite-P1 was observed. The sodalite and gismondine diffraction peaks increased in intensity with increasing Ms, and when Ms was increased to 1.6 (Ms 4), the zeolite-P1 diffraction peak disappeared and the intensity of the C_2_S diffraction peak increased. This was because when Ms was 1.0 or 1.2, excessive alkali in the system led to an excessively rapid reaction and the dissolved silica-aluminate gel in the early stage of the reaction covered the surface of the reaction phase, hindering the cross-linking of the gel-like network structure in the geometric system^27^, This in turn affected the formation and growth of zeolite crystal nuclei, resulting in incomplete crystal growth. When Ms was 1.6, insufficient alkalinity in the system meant that the silica-aluminate ions in the geometric were unable to fully dissolve, resulting in an incomplete reaction.

#### Curing time

**Figure 5:**
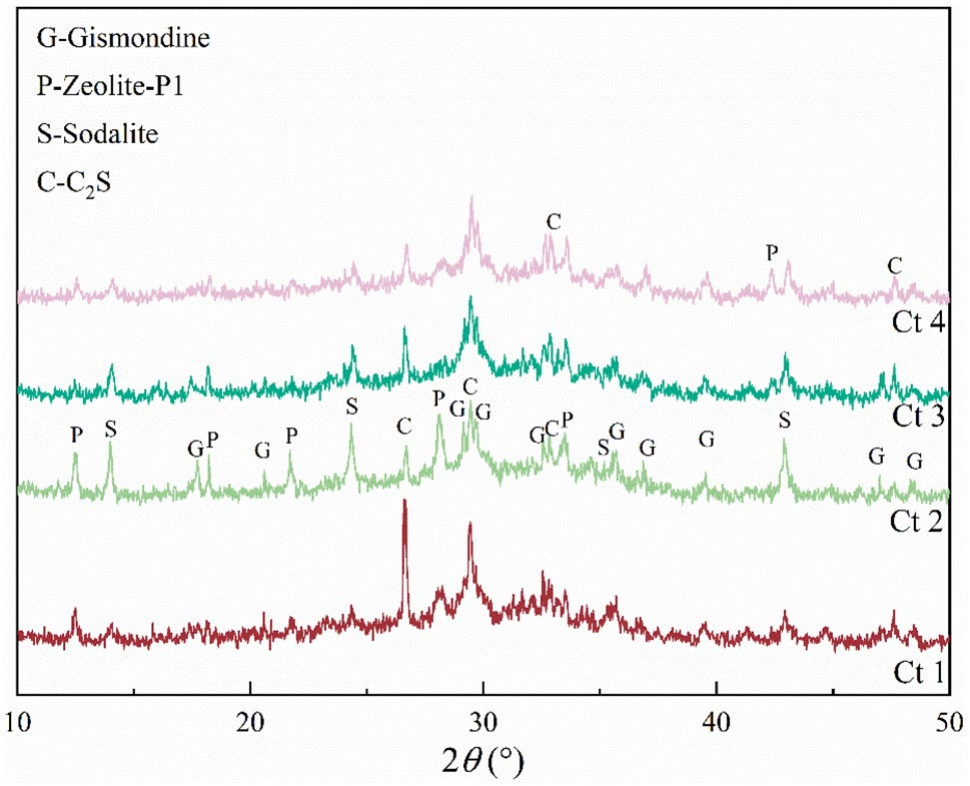
XRD patterns of hydrothermal products with different curing times; Ct 1: curing time at 6 h, Ct 2: curing time at 12 h, Ct 3: curing time at 18 h, Ct 4: curing time at 24 h.

The curing time of geopolymers significantly influences their microstructure and the subsequent formation of zeolite^27,28^. Figure 5 shows the XRD patterns of zeolites obtained with different geopolymer curing times. No zeolite phase was observed when the curing time was 6 h (Ct 1). The main component formed with a curing time of 6 h was C_2_S, whose diffraction peak was narrow and high. When the curing time was increased to 12 h (Ct 2), the diffraction peak of C_2_S was weakened and the distribution of the zeolite phase was significantly increased. This was potentially because an excessively short curing time meant that the silica-aluminate ions were not fully spread through the system, leading to an incomplete reaction. However, with increasing curing time, more aluminosilicate ions were involved in the reaction, promoting the production of the zeolite facies. When the curing time was longer than 12 h, the diffraction peaks of each phase were not affected, potentially indicating that the reaction was stabilized at this time. Thus, the composition and structure of the geopolymer gels remained relatively stable, so the mineral composition of the products after hydrothermal treatment did not change.

#### Curing temperature

**Figure 6:**
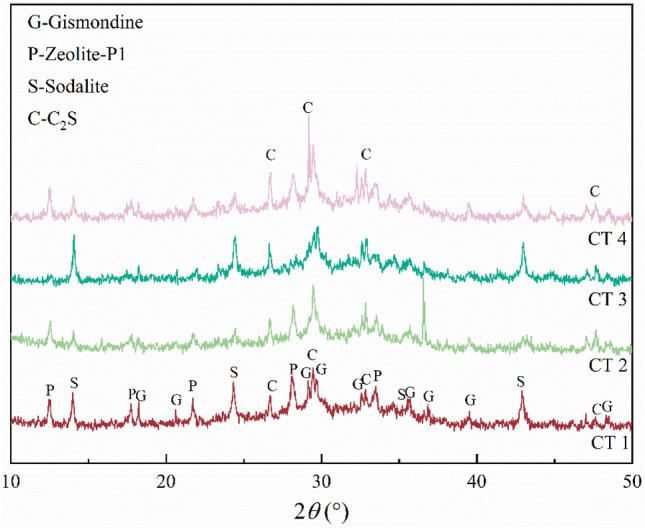
XRD patterns of hydrothermal products with different curing temperatures; CT 1: curing temperature it 40 °C, CT 2: curing temperature at 60 °C, CT 3: curing temperature at 60 °C, CT 4: curing temperature at 80 °C.

When preparing a geopolymer, curing temperature plays an important role in the geopolymer polymerization reaction, the nucleation of the zeolite molecular sieve, and crystal growth^29^. Figure 6 shows the XRD patterns of zeolites obtained with different curing temperatures. With increasing curing temperature, the C_2_S diffraction peak became stronger, while the zeolite phase diffraction peaks became weaker. This demonstrated that an excessively high curing temperature was not conducive to the formation of zeolite. This was potentially because the reaction rate increased and the silica-aluminate ions dissolution rate was higher when the reaction temperature was higher. Thus, more gel phases covered the surface of the dissolved LFS and FA, which hindered the dissolution of silica-alumina caused by the incomplete reaction and affected the formation of zeolite phases^30^.

#### Hydrothermal time

**Figure 7:**
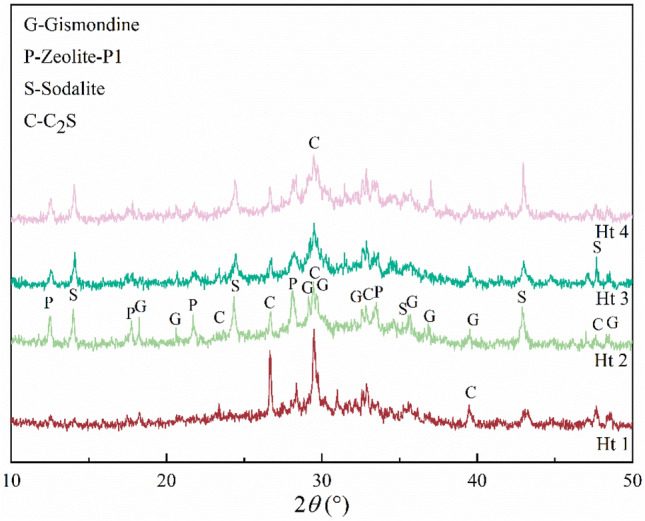
XRD patterns of hydrothermal products with different hydrothermal times; Ht 1: hydrothermal time at 6 h, Ht 2: hydrothermal time at 12 h, Ht 3: hydrothermal time at 18 h, Ht 4: hydrothermal time at 24 h.

Hydrothermal treatment time significantly influences the crystallinity of zeolites. An excessively short hydrothermal time means that a crystalline phase cannot be formed, and an excessively long hydrothermal time leads to the generation of a hybrid phase^28^. Figure 7 shows the XRD patterns of zeolites prepared with different hydrothermal times. When hydrothermal time was 6 h (Ht 1), the predominant product was C_2_S and no obvious zeolite phases were produced. This was likely because the hydrothermal time was too short, so the silica-aluminate ions failed to participate in the precursor depolymerization reaction. This time was potentially the induction period of zeolite formation, involving the gel formation of crystal nuclei. These conditions were insufficient for the formation of zeolite. Thus, a longer hydrothermal time was required to provide more activation energy to break the structure. When the hydrothermal time was increased to 12 h (Ht 2), the diffraction peak strength of C_2_S weakened, and the diffraction peak of the zeolite phases significantly increased in intensity. When the hydrothermal time was further extended to 24 h (Ht 4), the peak strength and peak type did not significantly change, indicating that the growth and dissolution of crystals reached dynamic equilibrium at this time^31^.

#### Hydrothermal temperature

**Figure 8:**
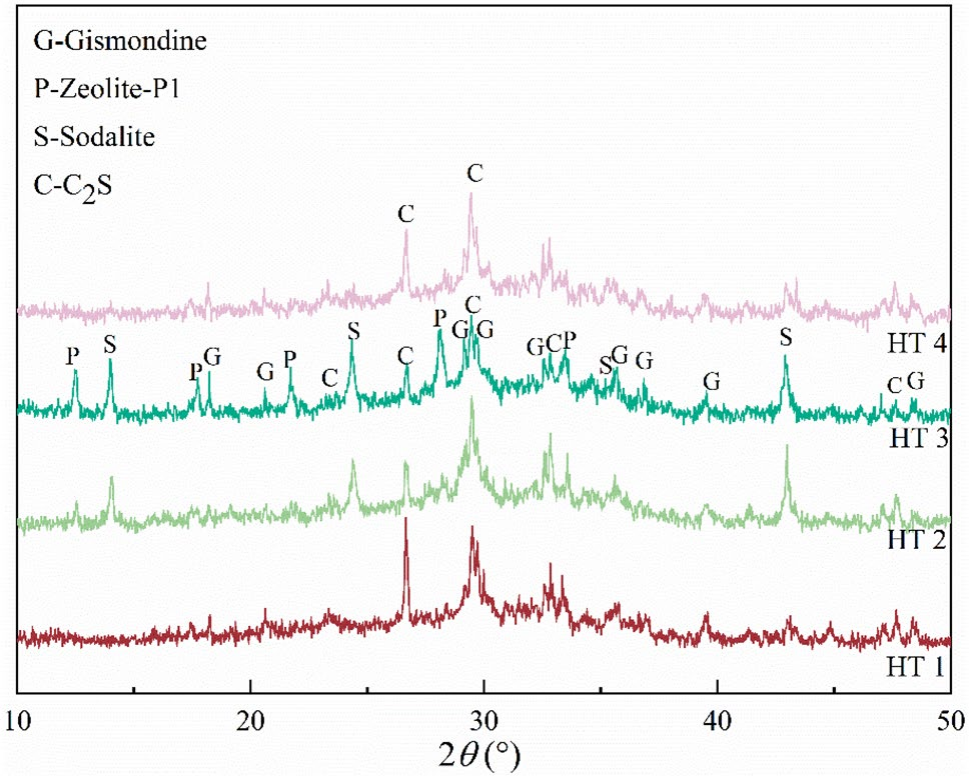
XRD patterns of hydrothermal product with different hydrothermal temperatures; HT 1: hydrothermal temperature at 60 °C, HT 2: hydrothermal temperature at 90 °C, HT 3: hydrothermal temperature at 120 °C, HT 4: hydrothermal temperature at 150 °C.

Hydrothermal temperature also has a significant influence on the formation of zeolite. Higher temperatures can improve the concentration of chemical groups in the sol, which is conducive to zeolite crystallization^31^. Figure 8 shows the XRD patterns of zeolites prepared at different hydrothermal temperatures. When the hydrothermal temperature was 60 °C (HT 1), no obvious zeolite phase was observed, and the main component was C_2_S. With increasing hydrothermal temperature, some zeolite phases such as sodalite and gismondine gradually appeared. When the temperature was increased to 120 °C (HT 3), the diffraction peak of zeolite-P1 appeared, although it disappeared at 150 °C (HT 4). This was similar to the research results of^32^, that is, the suitable hydrothermal temperature for obtaining zeolite-P1 was 120 °C. This was potentially because an excessively low hydrothermal temperature was not sufficient for zeolite nucleation, and there was not enough energy to break the barrier for C_2_S and other inert substances. With increasing hydrothermal temperature, the crystal structures of these inert substances were attacked and destroyed, releasing more Si and Al leading to the gradual formation of [SiO_4_]^4−^ and [AlO_4_]^5−^ tetrahedra under strong alkali conditions. Moreover, increasing the hydrothermal temperature also enhanced the movement rate of silica-aluminate ions, increased the effective collision rate, and accelerated the formation of the silicon-aluminate gel. This allowed more of the silicon-aluminate ions to be involved in the reaction, which in turn led to the generation of more zeolite phases. However, When the temperature was increased to 150 °C, more dissolved Ca^2+^ was present in the reaction system, which inhibited zeolite formation^17,33^.

#### Characterization of zeolite prepared under optimal conditions

According to the experimental analysis reported in Section “Influence of synthesis conditions on zeolite”, the optimal synthesis conditions for the preparation of the molecular sieve are as follows: an LFS to FA ratio of 4:6, a hydrothermal time of 12 h, a hydrothermal temperature of 120 °C, Ms = 1.4, a NaOH concentration of 4 mol/L, a curing temperature of 40 °C, and a curing time of 12 h. The phase composition, microstructure, functional group changes, and pore size distribution of the zeolite prepared under these reaction conditions were analyzed, and the formation mechanism of the zeolite molecular sieve was described through the change of Si–Al.

#### Changes in functional groups during zeolite synthesis

**Figure 9:**
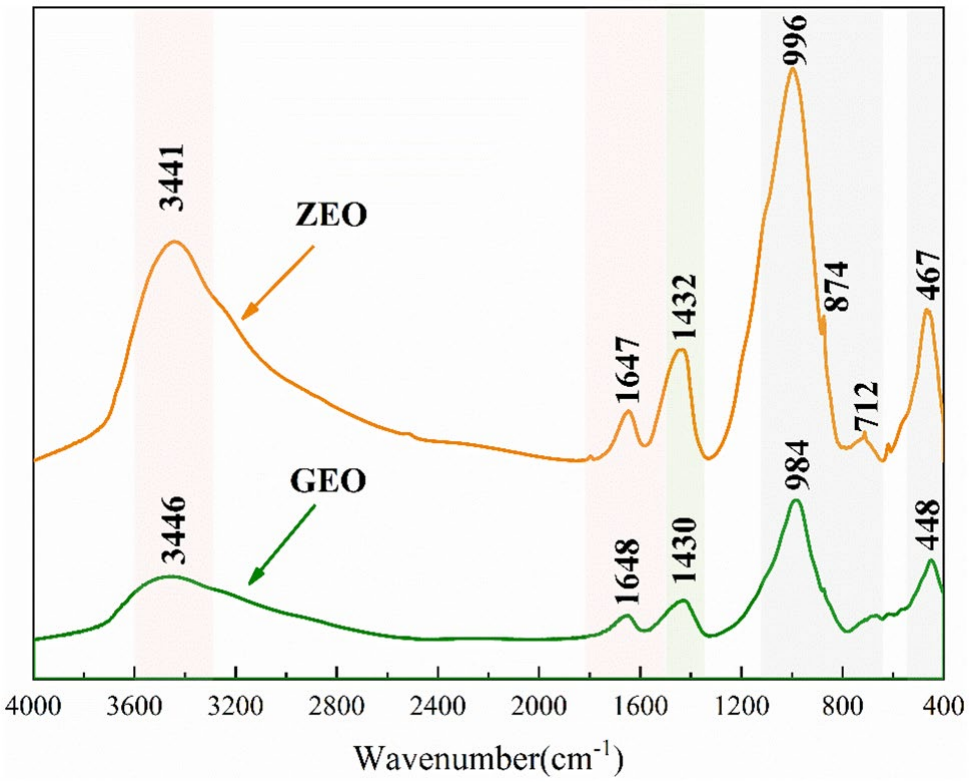
The infrared spectra of the geopolymer (GEO) and the final product (ZEO).

Figure 9 shows the infrared spectra of the geopolymer (GEO) and the final product (ZEO). The vibration peaks of GEO and ZEO at 3500–3400 cm^−1^ and 1700–1600 cm^−1^ are the stretching and bending vibrations of O–H in water^34^, respectively. These bands indicate the presence of water molecules in GEO and ZEO, which plays a role in the transmission and diffusion of silica-aluminate ions during the zeolite formation process. The peak at 1500–1400 cm^−1^ is the C–O tensile vibration of carbonate^35^, which may be caused by the reaction of NaOH with the Ca-based components in LFS and CO_2_ in the air^34^. The strong vibration peak at 1000–900 cm^−1^ is the asymmetric stretching peak of Si–O–T (T = Si, Al) in the TO_4_ tetrahedral structure. The vibration peak at 500–400 cm^−1^ was ascribed to the T–O bending vibration peak of the inner TO_4_ tetrahedron^34,36^. Compared with GEO, two additional vibration peaks were observed in the ZEO spectrum after hydrothermal treatment. These were the vibration peak at 874 cm^−1^, which was the stretching vibration of Si–O–Si, and the symmetric stretching of zeolite tetrahedron at 712 cm^−1^, which is one of the characteristic peaks of zeolite molecular sieves^13,37^. At the same time, it was observed that after hydrothermal treatment, the peak at 984 cm^−1^ shifted to 996 cm^−1^, which was caused by the increasing degree of active aluminum cross-linking and the degree of silico-aluminate gel polymerization^38^. This indicated that silicon and aluminum were further aggregated to form zeolite structures with a higher degree of polymerization and cross-linking during the hydrothermal process.

#### Phase composition and microstructure changes of synthesized zeolite molecular sieve

**Figure 10:**
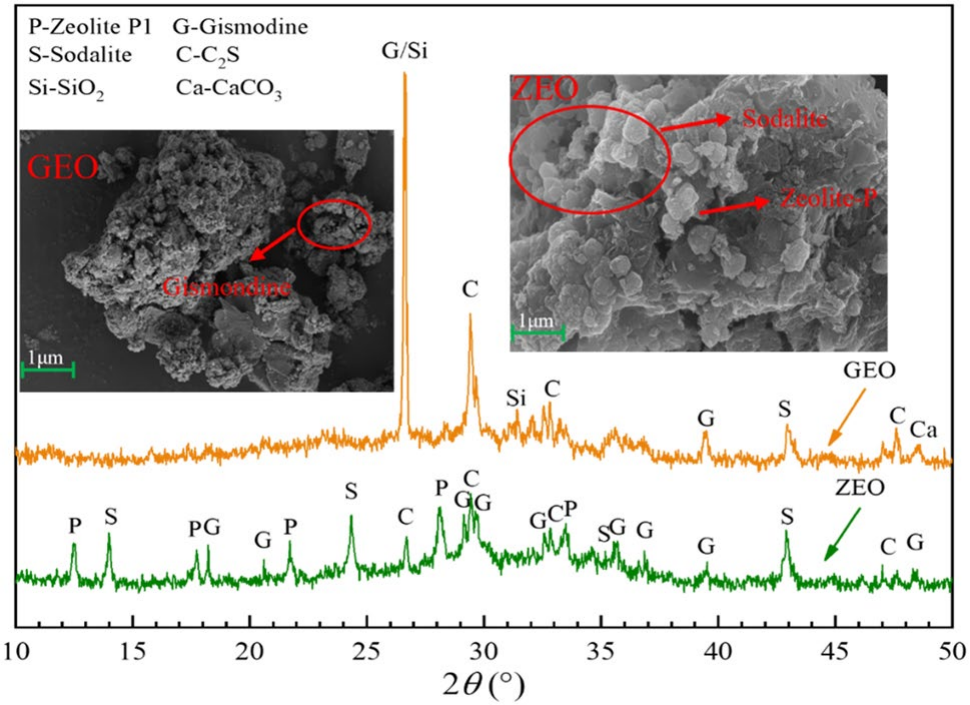
XRD and SEM imagines of GEO and ZEO; P: zeolite P1, G: gismodine, S: sodalite, C: C_2_S, Si: SiO_2_, Ca: CaCO_3._

Figure 10 shows XRD and SEM images of GEO and ZEO. After hydrothermal treatment, the SiO_2_ peak disappeared, the intensity of the C_2_S diffraction peak decreased, and a zeolite phase diffraction peak appeared. This indicates that the C_2_S and SiO_2_ lattice in the GEO was destroyed by NaOH attack at a high temperature (120 °C), upon which it dissolved at the surface of the reaction phase and released Ca^2+^ and Si^4+^ ions. These ions subsequently reacted with OH^-^ and water molecules in the liquid phase to form the zeolite phase. The SEM analysis showed that the GEO was a mostly amorphous silica-aluminate gel with a small amount of needle and rod-shaped gismondine covering the smooth and dense surface. The ZEO after hydrothermal treatment was mostly sodalite with spherical morphology and a small amount of zeolite-P1 with a cubic morphology covering the surface of the reaction phase. This was in good agreement with the XRD results, demonstrating the production of a zeolite phase.

#### Changes in pore size distribution and pore structure of synthetic zeolite molecular sieves

**Figure 11:**
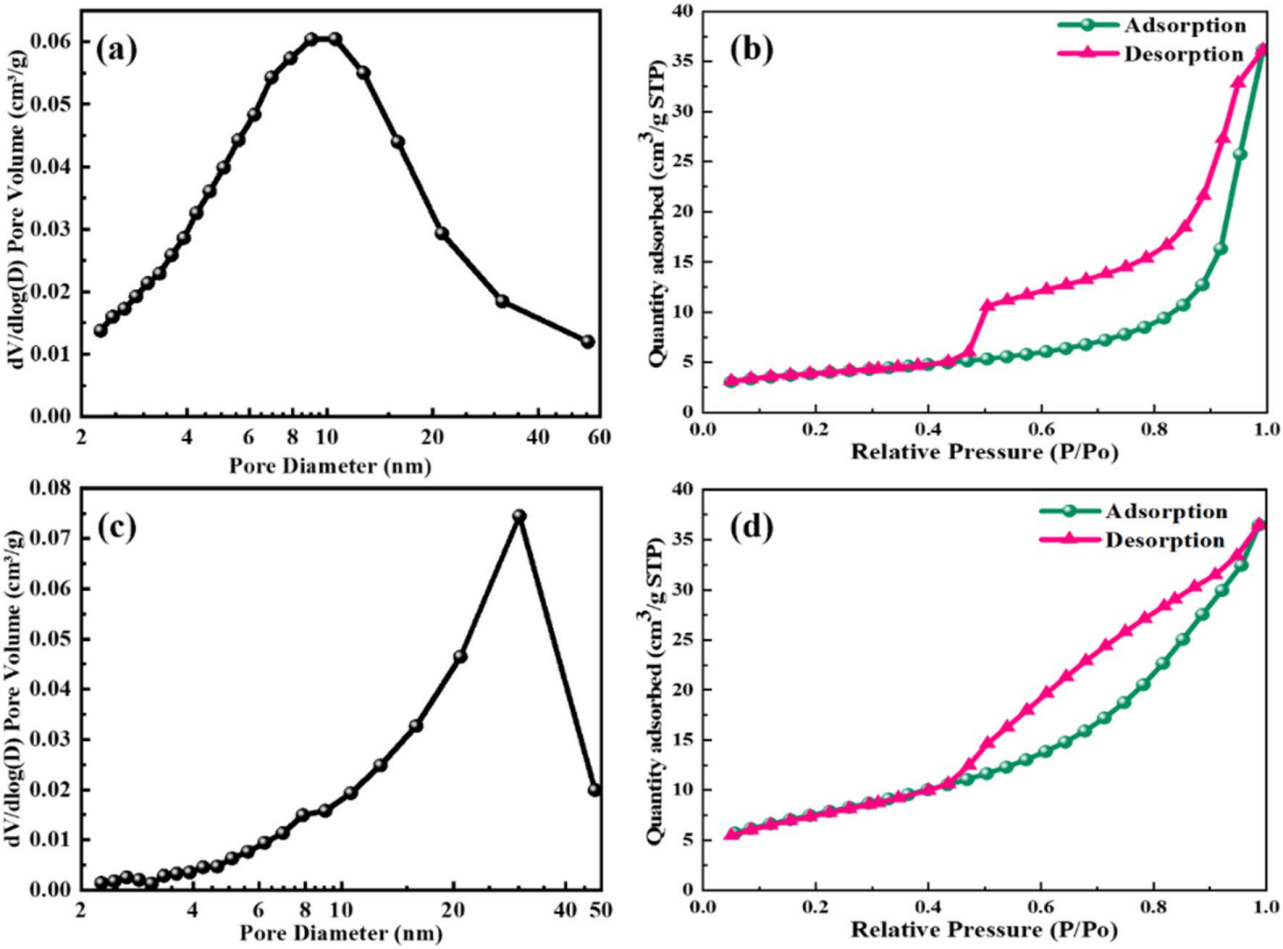
(**a**) The pore size distributions of GEO; (**b**) The Nitrogen adsorption–desorption isotherms of GEO; (**c**) The pore size distributions of ZEO; (**d**) The Nitrogen adsorption–desorption isotherms of ZEO.

Table 3 shows the texture parameters of GEO and ZEO, Fig. 11 shows the N_2_ adsorption–desorption isotherms and particle size distributions of GEO and ZEO. Both GEO and ZEO showed type IV adsorption–desorption isotherms with an H3 type hysteresis loop. Thus, these materials were mesoporous (with pores in the range of 2 to 50 nm) and their mesopores were mainly slit pores. Moreover, this type of hysteresis loop is mainly typical of the non-rigid aggregation of lamellar particles. Capillary coalescence occurred at a P/P_o_ of 0.43, which was due to the mesopores generated by inter-particle stacking^39^. The pore size of GEO was concentrated in the range of 0–30 nm, while that of ZEO was concentrated in the range of 20–40 nm, indicating that hydrothermal treatment increased the pore size. This was consistent with the SEM analysis.

**Table 3:** Texture parameters of GEO and ZEO.

Specimen	Pore volume (cm^3^/g)	BJH adsorption average pore diameter (nm)	Surface area(m^2^/g)	
			S_BET_	S_t-plot_
GEO	0.052	52.3	13.1	2.46
ZEO	0.050	167.0	27.3	28.66

#### Analysis of the mechanism of zeolite molecular sieve synthesis from LFS-FA geopolymer

**Figure 12:**
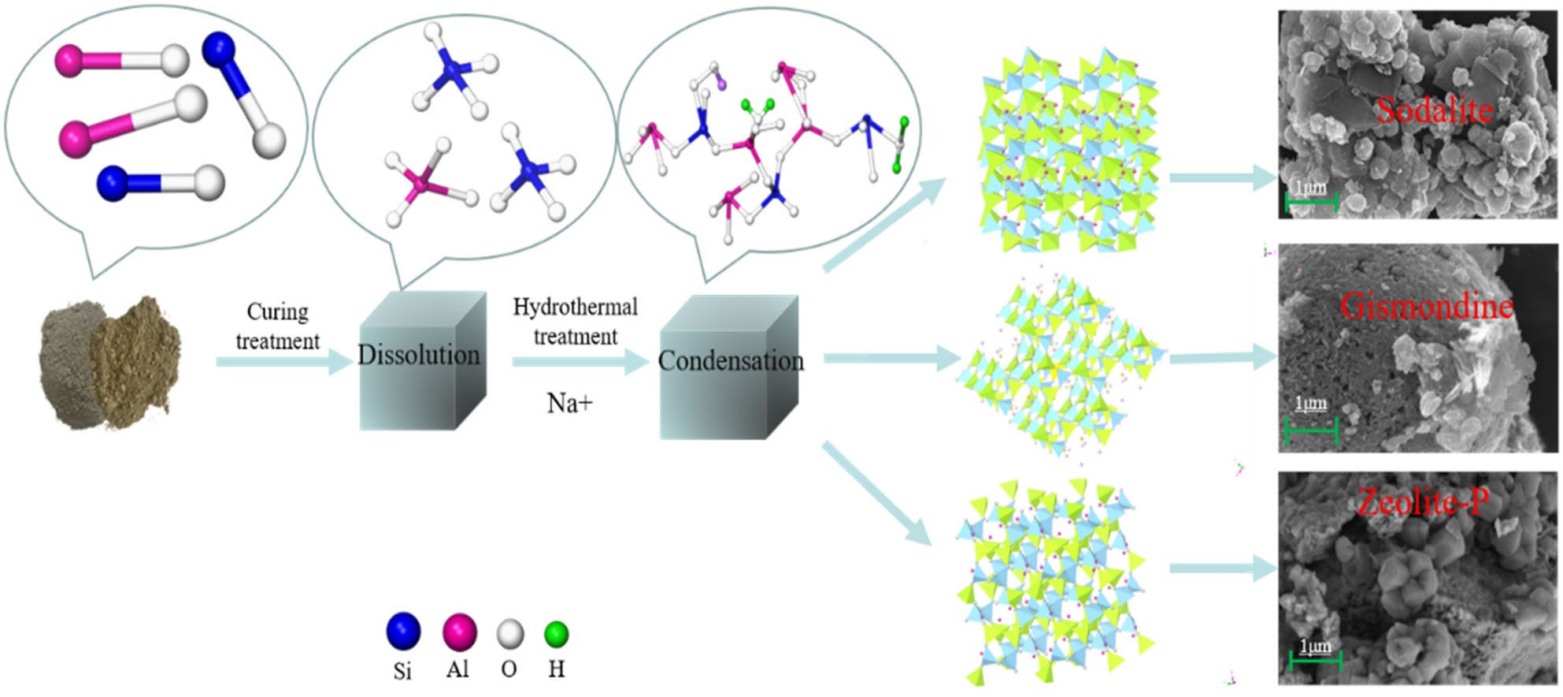
The Mechanism formation of zeolites.

Figure 12 shows the formation mechanism of the zeolite. During this process, Si and Al in the LFS and FA are first dissolved to form [SiO_4_]^4−^ and [AlO_4_]^5−^ tetrahedra on the surface of the reaction phase under the action of Sodium silicate excitation. At this point, water acts as the main diffusion transport medium. but as the liquid–solid ratio are relatively low, the tetrahedra are difficult to diffuse and therefore only crosslink randomly with the surrounding tetrahedral structure to form an amorphous silica-aluminate gel^9^. This provides a large enough cavity to accommodate charge-balanced alkali ions^40^. This stage includes gel formation and solidification due to condensation and the formation of 3D silicaluminate networks, in which the sodium silicate modulus, curing time, and curing temperature have a significant effect on the structure and rate of silica-aluminate gel formation^25–27^. The resulting geopolymer gel has a zeolite-like structure. During the high-temperature hydrothermal process, silica-aluminate gel will release [SiO_4_]^4−^ and [AlO_4_]^5−^ tetrahedra under high temperature and attack by NaOH, at the same time, the anions do not work in isolation, but the driving force of diffusion is enhanced by the structural orientation of Na^+^ and the diffusive transport of water. Moreover, cations such as Na^+^ and Ca^2+^ attract [SiO_4_]^4−^ and [AlO_4_]^5−^ tetrahedra around themselves^28^, causing the disordered tetrahedra to rearrange and further cross-link and condense on the surface of the particles to form a regularly arranged tightly packed zeolite network structure. After hydrothermal treatment, the products cover the surface of the reaction phase, so it can be inferred that the reaction occurs on the surface of the reaction phase and that these products inhibit the further dissolution of Si and Al within the particles, resulting in an incomplete reaction. This also explains why C_2_S is still present after hydrothermal treatment.

In addition, the bond energies of Si–O, Al–O, and Ca–O are 444, 221–280, and 134 kJ/mol, respectively^41^, and the bond energy of Ca–O bond is the weakest. Therefore, Ca–O is preferentially broken under alkali activation conditions, releasing Ca^2+^. With the rapid release and accumulation of excess Ca^2+^, a calcium-rich silica-aluminate gel is preferentially formed and precipitated on the surface of the reaction phase, hindering the release of Si^4+^ and Al^3+^. In turn, this causes an incomplete reaction.

## Conclusion

In this paper, products with zeolite-P1, sodalite, and gismondine phases were successfully formed by in situ hydrothermal method after the preparation of a geopolymer from LFS and FA without treatment (acid leaching, alkali melting etc.). The optimal preparation conditions for preparing the best zeolite molecular sieve were an LFS:FA ratio of 4:6, a NaOH concentration of 4 mol/L, a sodium silicate modulus of 1.4, a hydrothermal temperature of 120 °C, a hydrothermal time of 12 h, a curing temperature of 40 °C, and a curing time of 12 h. The degree of polymerization and cross-linking of the silica-aluminate gel increased after hydrothermal treatment, resulting in the formation of a zeolite structure. The hydrothermal products were mesoporous, showing good promise for use as adsorbent materials.

During the reaction, the silica-alumina ions in the LFS and FA were excited by the alkali exciter to form an indeterminate silica-aluminate gel with a tetrahedral structure. Subsequently, during the high-temperature hydrothermal process, diffusion was enhanced by the structural orientation of Na^+^ and the diffusive transport of water. Then, the [SiO_4_]^4−^ and [AlO_4_]^5−^ tetrahedra were rearranged and condensed around the free Ca^2+^ and Na^+^ to form the zeolite structure. In addition, excess Ca^2+^ led to the preferential formation and precipitation of this silica-aluminate gel and adhered to the surface of the reaction phase, impeding further reaction. The mesoporous structure of the products may have the potential to be used for heavy metals and impurities of waste water as well as gases such as CO_2_.

## Data Availability

The datasets generated during and/or analyzed during the current study are available on resonable request, by contacting the corresponding author; Yuanrong Yi(yyrhyw@163.com).
